# The role of microRNAs in nasopharyngeal carcinoma

**DOI:** 10.1007/s13277-014-2847-3

**Published:** 2014-11-27

**Authors:** Gongjun Tan, Xiaowei Tang, Faqing Tang

**Affiliations:** 1 Medical Research Center, Xiangya Hospital, Central South University, 87 Xiangya Road, Changsha, 410008 Hunan China; 2 Department of Clinical Laboratory, Zhuhai Hospital, Jinan University, Zhuhai, 519000 Guangdong China; 3 2011 Undergraduate, Metallurgical Science and Engineering, Central South University, 923 Lushan South Road, Changsha, 410083 China

**Keywords:** microRNA, Nasopharyngeal carcinoma, Tumorigenesis, Metastasis, Biomarker, Therapeutic target

## Abstract

Nasopharyngeal carcinoma (NPC), a distinct type of head and neck cancer, is prevalent in Southeast Asia and southern China. Ethnic background and environmental factors contribute to the development of NPC, further complicating its pathogenesis. An increasing body of evidence indicates that microRNAs (miRNAs) play an important role in the development and progression of NPC, in particular, 32 miRNAs are involved in NPC tumorigenesis, progression, and metastasis. The causal involvement of miRNAs in NPC and their possible use as biomarkers have been extensively studied with promising results, demonstrating the diagnostic and therapeutic potential of miRNAs in NPC. In this review, we summarize the role of all the known miRNAs involved in the signaling pathway implicated in NPC.

## Introduction

Nasopharyngeal carcinoma (NPC) is endemic in southern China and Southeast Asia with an annual incidence of 15–50 cases per 100,000 [1]. Both ethnic background and environmental factors contribute to the risk of NPC development. The Chinese population emigrating to Southeast Asia or North America is considered to be at high risk. In contrast, the incidence of NPC is much lower in the Chinese individuals born in North America [2, 3]. According to global cancer statistics reported by the International Agency for Research on Cancer, over 84,000 new NPC cases occur annually, among which 80 % are located in Asia and 5 % in Europe. NPC is characterized by poorly or undifferentiated carcinoma. It differs from non-nasopharyngeal head and neck squamous cell carcinomas in several ways including its association with the Epstein–Barr virus (EBV) and a great propensity for distant metastases [4]. Advances in radiotherapy and comprehensive chemotherapy strategies have greatly improved outcomes in the patients with primary NPC; the 5-year survival rate increases from 50 % in the 1980s to 70 % in the 1990s [5, 6]. However, 15–58 % of NPC patients experience recurrence of the disease and have to undergo re-treatment [7, 8].

MicroRNAs (miRNAs or miRs) are endogenous, small, non-coding single-stranded RNAs of ∼22 nucleotides in length, of which negative regulators of gene expression are small non-coding RNAs that typically inhibit the translation and reduce the stability of messenger RNAs (mRNAs). Thereby, they are involved in cellular processes such as inflammation, cell-cycle regulation, stress response, differentiation, apoptosis, and migration [9]. The annotation of miRNAs genomic positions indicates that most miRNA-coding genes are located in the introns of protein-coding genes, and the introns or exons of non-coding genes [10]. miRNAs can be organized as individual genes or localized as clusters representing miRNA families, which are commonly related in their sequence and function. miRNAs are mainly transcribed by RNA polymerase II (RNA pol II) from their own promoter or the promoter of the host gene in which they reside. The RNA pol II synthesizes large miRNA precursors called primary-miRNAs (pri-miRNAs) [11]. The canonical miRNA biogenesis pathway consists of two main processing steps that occur in the nucleus and the cytoplasm. pri-miRNAs are first processed into the nucleus and then cleaved into a 60–70 nt double-helix hairpin structure (called precursor miRNA or pre-miRNA) by the RNase III Drosha and its cofactor DGCR8 [12]. Exportin 5 mediates the transfer of the pre-miRNA into the cytoplasm [13], where the second cropping process (dicing) takes place. The pre-miRNA is first processed by the RNase III Dicer, in concert with TAR RNA binding protein or protein activator of the interferon-induced protein kinase cofactors, into a 22 nt dsRNA with two-nucleotide 3′-overhangs called miRNA/miRNA* duplex. It is finally unwound by a cytoplasmic helicase [14]. The mature miRNA guide is generally selected according to its thermodynamic properties, while the complementary passenger strand is usually subject to degradation. It was formerly thought that the miRNA strand is preferentially degraded; however, more recent evidence suggests that it does not simply represent a non-functional byproduct of miRNA biogenesis but can be selected as the functional strand to play significant biological roles [15].

The miRNA strand with the less stable 5′ end (guide strand) is preferentially selected and incorporated in the RNA-induced Silencing Complex. The mature miRNA regulates gene expression at the posttranscriptional level by binding through partial complementarity to target mRNAs [generally the 3′-untranslated regions (UTR)], which results in mRNA degradation or translation inhibition [16]. Indeed, miRNAs mainly recognize complementary sequences in the 3′-UTRs of their target mRNAs; however, recent studies have reported that miRNAs can also bind to the 5′-UTR or the open reading frame. Surprisingly, they can also upregulate translation under conditions of growth arrest [17, 18]. Moreover, miRNAs, as well as dsRNAs, are known to bind promoter regions at the genomic level and induce gene expression by returning to the nucleus or a hexanucleotide terminal motif-mediated transfer [19, 20].

Genetic variants of the miRNA biogenesis pathway are associated with the risk and/or survival in various malignancies such as bladder, ovarian, prostate, and breast cancer [21–24]. miRNAs can also be secreted in the surrounding region or the biological fluids where they are protected in liposomal-like particles and act on other cell types. Indeed, increasing evidences reveal that circulating miRNAs are associated with microvesicles—small exosomes/vesicles of endocytic origin, which are released by normal healthy or damaged cell types. In the present review, we summarize miRNAs in the carcinogenesis and metastasis of NPC, their potential as therapeutic targets and diagnostic markers, and their mediating signal pathway. This provides a novel clue for further investigating NPC.

## miRNAs are involved in multiple stages of NPC tumorigenesis

### miRNA expression in NPC

Since the discovery of miRNAs and their involvement in chronic lymphocytic leukemia [25], the role of miRNA dysregulation in NPC pathogenesis has been studied extensively [26]. miRNAs may function as oncogenes or tumor suppressors in the malignant progression of different tumor types. Profiling analysis indicated that a large number of miRNAs are either down- or upregulated in NPC tissues. Table 1 enlists a number of miRNAs involved in NPC, as supported by validated experimental data.

**Table 1:** List of miRNAs involved in NPC, their validated targets, and biological functions

miRNAs	Validated target(s)	Main biological function(s)
Tumor suppressors		
miR-1	PTMA	Induces carcinoma cell apoptosis
miRNA let-7	c-Myc, EZH2	Inhibits cell proliferation and induces cell apoptosis
miR-9	CXCR4	Regulates proliferation, EMT, invasion, metastasis, apoptosis, and tumor angiogenesis
miR-26a	EZH2, c-Myc	Suppresses cell proliferation and colony formation
miR-29c	TIAM1	Inhibits cell migration and invasion
miR-98	EZH2	Inhibits relapse
miR-124	Foxq1	Inhibits cell growth, migration, and invasion
miR-138	CCND1	Suppresses cell proliferation and colony formation
miR-184	BCL2, c-Myc	Suppresses cell proliferation
miR-200	ZEB2, CTNNB1, Notch1	Regulates EMT, migration, and invasion
miR-204	Stat-3, CDC42	Regulates invasion
miR-216b	PKCá, K-Ras	Suppresses proliferation and invasion
miR-375	MTDH	Suppresses relapse
miR-451	MIF	Regulates NPC cell growth and invasion
Onco-miRNAs		
miR-10b	MMP-9	Promotes mobility and invasion
miR-18a	Dicer1, c-Jun, c-Myc,	Lymph node metastasis
miR-18b	CTGF	Promotes cell growth
miR-21	BCL2	Promotes migration and proliferation
miR-30a	E-cadherin	Increases the capability of metastasis and invasion
miR-93	TGFâR2	Promotes cell proliferation, invasion, and metastasis
miR-141	BRD3, PTEN, SPLUNC1	Promotes cell growth, migration, and invasion
miR-144	PTEN	Promotes migration and invasion
miR-149	E-cadherin	Promotes mobility and invasion
miR-155	JMJD1A, BACH1	Stimulates cell proliferation, colony formation, cell migration, and invasion
miR-205	PTEN	Attenuates cell apoptosis post-irradiation
miR-214	LTF, Bim	Promotes NPC cell proliferation, invasion, and metastasis
miR-378	TOB2	Promotes cell proliferation, colony formation, migration, and invasion
miR-421	FOXO4	Induces cell growth and apoptosis resistance
miR-663	p21	Promotes cellular G1/S transition

*PTMA* prothymosin alpha or ProT alpha, *IFN* interferon, *MHC* major histocompatibility complex, *CXCR4* chemokine (C-X-C motif) receptor 4, *CCND1* cyclin D1, *ZEB* E-box binding homeobox, *CTNNB1* catenin (cadherin-associated protein), beta 1, *CDC42* cell division cycle 42, *PKC* protein kinase C, *MIF* macrophage migration inhibitory factor, *MMP-9* matrix metalloproteinase-9, *CTGF* connective tissue growth factor, *TGFβR2* transforming growth factor-β receptor II, *BRD3* bromodomain containing 3, *PTEN* phosphatase and tensin homolog, *SPLUNC1* short palate, lung, and nasal epithelium clone 1, *JMJD1A*, *BACH1* BTB and CNC homology 1, *LTF* lactotransferrin, *TOB2* transducer of ERBB2, *FOXO4* forkhead box O 4

### miRNA functions as a tumor suppressor to regulate NPC

**Fig. 1:**
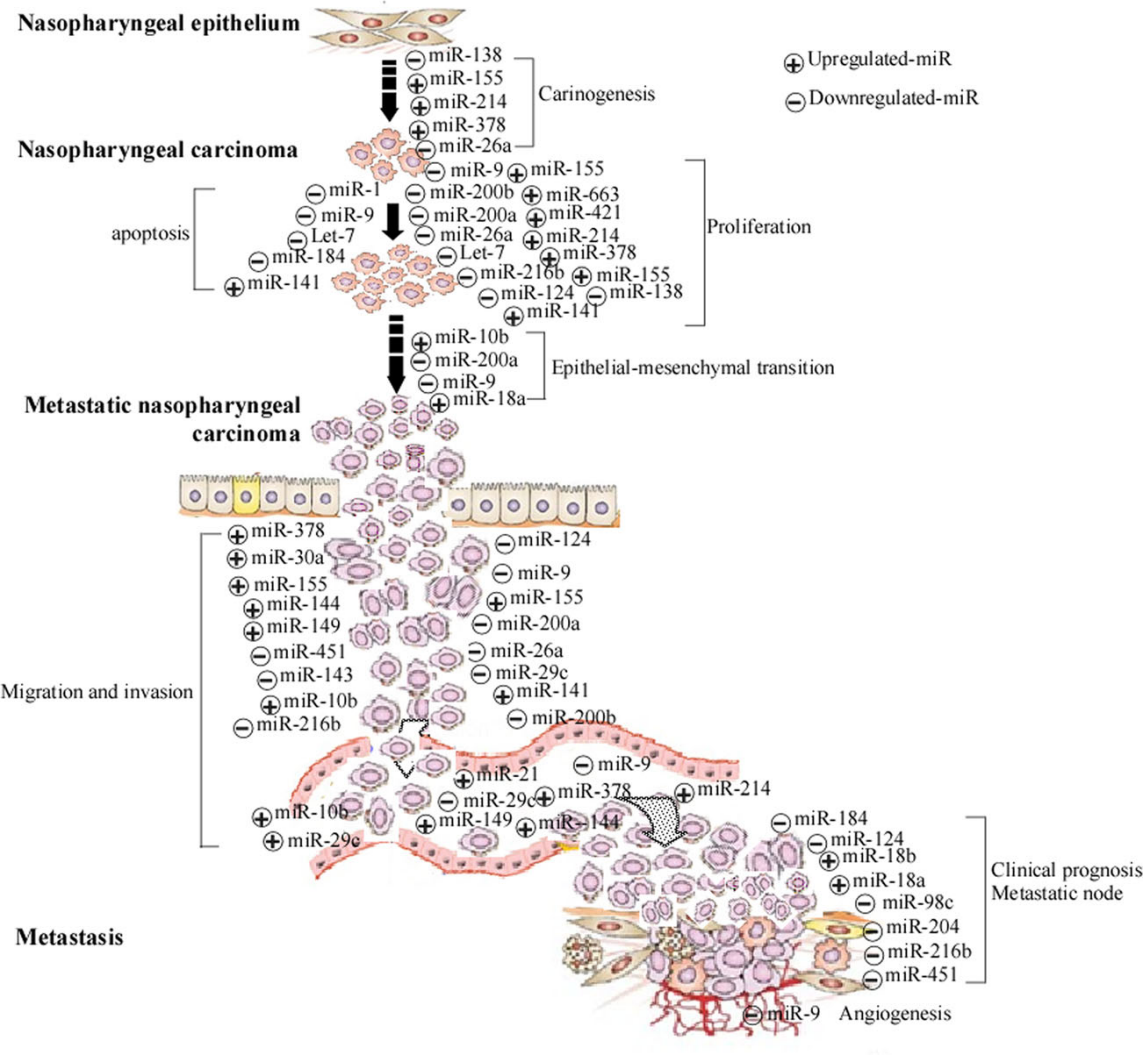
Schematic illustration of microRNAs (miRNA) involved in nasopharyngeal carcinoma (NPC). miRNAs are involved in nasopharyngeal epithelium carcinogenesis, NPC cell apoptosis, proliferation epithelial–mesenchymal transition, migration, and invasion. Some miRNAs are associated with clinical prognosis. *Plus sign* upregulated miRNA; *minus sign* downregulated miRNA

Generally, cancer develops sophisticated networks of multiple signaling pathways, which contribute to their ability to progress, in some cases, and to evade treatment. Gain-of-function and loss-of-function experiments, in combination with target prediction analyses, have demonstrated that miRNAs can influence multiple steps of tumorigenesis [27]. miRNAs that function as tumor suppressors are often downregulated in NPC tissues. Since these miRNAs are negative regulators of protein-coding genes, the downregulation of miRNAs is expected to cause an upregulation of their target genes and subsequent alterations of the associated cellular pathways in NPC (Fig. 1). Among the tumor suppressor microRNAs involved in NPC, the miR-9, miR-26, miR-29, miR-200 family, and the Let-7 family are the most common.

miR-9 appears to be one of the most important players in NPC biology; while it is commonly downregulated in NPC, it is known to regulate proliferation, epithelial–mesenchymal transition (EMT), invasion, metastasis, apoptosis, and angiogenesis in other cancers (Fig. 1) [28, 29]. Mechanistically, miR-9 can directly bind to the 3′-UTR of CXCR4 to downregulate its expression. CXCR4 downregulation inhibits cell growth, migration, and invasion, whereas its overexpression rescues the suppressive effect of miR-9 [30]. In addition, miR-9 can modulate the expression of interferon-induced genes and major histocompatibility complex class I molecules in human cancer cells, indicating a novel role of miR-9 in linking inflammation and cancer [31]. Ectopic expression of miR-9 dramatically inhibits the proliferative, migratory, and invasive capabilities of NPC cells both in vitro and in vivo. Low plasma levels of miR-9 are significantly correlated with increased lymphatic invasion and advanced TNM stage. Furthermore, these levels are significantly higher in post-treatment samples than in pre-treatment samples [32]. miR-26a functions to suppress growth in NPC; however, miR-26a is commonly downregulated in NPC specimens and cell lines; its ectopic expression dramatically suppresses cell proliferation and colony formation by inducing G_1_ cell-cycle arrest. In addition, miR-26a strongly reduces the expression of the EZH2 (Zeste homolog 2) oncogene in NPC cells (Fig. 1) [33]. Mechanistically, miR-26a suppresses the expression of c-Myc, cyclins D3 and E2, and the cyclin-dependent kinases (CDK), CDK4 and CDK6, enhances the expression of the CDK inhibitors p14(ARF) and p21(CIP1) in an EZH2-dependent manner. The antimetastatic functions of miR-26a are primarily mediated by repressing EZH2 expression [34]. miR-29c is also downregulated in NPC; its ectopic expression inhibits NPC cell migration and invasion in vitro and suppresses the formation of lung metastases in vivo. Studies using luciferase reporter assays have confirmed that T cell lymphoma invasion and metastasis 1 (*TIAM*1) is a miR-29c target gene [35]. Reduced expression of miR-29c is positively correlated with therapeutic resistance in NPC patients. Mechanistically, miR-29c substantially enhances the sensitivity of NPC cells to ionizing radiation (IR) and cisplatin treatment through the repression of antiapoptotic factors, Mcl-1 and Bcl-2 [36]. Moreover, miR-98 is also found to be underexpressed in relapsed NPC patient samples. Using luciferase-based assays, miR-98 has been validated as a bona fide regulator of the expression of EZH2, which is associated with a higher risk of relapse in NPC patients [37].

miR-200 family is often downregulated in NPC. The overexpression of miR-200a inhibits the growth, migration, and invasion of C666-1 cells (Fig. 1), whereas its knock-down stimulates these processes in CNE-1 cells. It is speculated that E-box binding homeobox 2(ZEB2), catenin (cadherin-associated protein), and beta 1(CTNNB1) are the functional downstream targets of miR-200a. The knock-down of ZEB2 solely impedes NPC cell migration and invasion, whereas CTNNB1 suppression inhibits NPC cell growth. These suggest that the inhibitory effects of miR-200a on NPC cell growth, migration, and invasion are mediated by distinct targets and pathways [38]. miR-200a also regulates EMT as well as stem cell-like transition in NPC cells (Fig. 1). Stable knockdown of miR-200a promotes the transition of epithelium-like CNE-1 cells to the mesenchymal phenotype. Similarly, stable overexpression of miR-200a transfers the mesenchyme-like C666-1 cells to the epithelial state along with a significant reduction of their stem cell-like features. Mechanistically, miR-200a controls EMT by targeting ZEB2, and it also regulates the stem cell-like transition differentially and specifically through β-catenin signaling [39]. EBV-encoded EB nuclear antigen 1 (EBNA1) inhibits the expression of miR-200a and miR-200b, which results in the upregulation of target genes expression and zinc finger E-box binding homeobox 1 (ZEB1) and ZEB2 [40]. miR-200b is significantly downregulated in the NPC tissues and cell lines. Gain-of-function and loss-of-function studies have demonstrated that miR-200b suppresses NPC cell growth, migration, and invasion in vitro (Fig. 1). Importantly, the overexpression of miR-200b effectively represses tumor growth in nude mouse models. Furthermore, integrated analysis has identified Notch1 as a direct and functional target of miR-200b. Overexpression of Notch1 reverses the inhibitory effect of miR-200b on NPC cell growth and invasion [41].

The Let-7 family is one of the best known miRNA families in cancer biology. It regulates numerous oncogenes like RAS, MYC, and high-mobility group AT-hook 2 (HMGA2) [42, 43]. NPC cells exhibit reduced levels of miRNA Let-7 (Let-7-a, Let-7-b, Let-7-d, Let-7-e, Let-7-g, and Let-7-i). Ectopic expression of the Let-7 family inhibits NPC cell proliferation by downregulating c-Myc expression. Demethylation treatment of NPC cells causes the activation of Let-7 expression in poorly differentiated NPC cells [44]. EZH2 is a direct target of Let-7a, and the in vitro inhibition of EZH2 by Let-7a and/or EZH2 siRNA attenuates NPC cell growth, inhibits cell proliferation, and induces cell apoptosis, respectively (Fig. 1) [45].

Besides the afore-mentioned microRNAs, miR-204, miR-216b, miR-143, miR-375, miR-451, miR-375, miR-1, miR-124, miR-138, and miR-184 are known to be downregulated in NPC cells and tissues (Fig. 1). Reduced expression of miR-204 is strongly associated with a more aggressive and poor prognostic phenotype of NPC. EBV-encoded latent membrane protein 1 (LMP-1) suppresses miR-204 expression by activating Stat-3 and enhances cell division cycle 42 (CDC42) activity to facilitate the invasion of NPC [46]. Similarly, decreased miR-216b expression is directly related to advanced clinical stage and lymph node metastasis in NPC. Both in vitro and in vivo assays have revealed that miR-216b attenuates NPC cell proliferation, invasion (Fig. 1), and tumor growth in nude mice. miR-216b exerts its tumor suppressor function by inhibiting the KRAS-related (protein kinase B) AKT and extracellular signal-regulated kinase (ERK) pathways [47]. Additionally, the miR-216b-mediated downregulation of protein kinase C α (PKCα) suppresses the proliferation and invasion ability of CNE2 cells; moreover, overexpressed PKCα can partially reverse the inhibitory effect of miR-216b on cell proliferation [48]. Patients with low miR-451 expression have poor overall and disease-free survival compared with those with high miR-451 expression. Macrophage migration inhibitory factor, a direct target of miR-451, regulates NPC cell growth and invasion [49]. miR-143 plays a role in modulating the invasiveness and metastasis of NPC; its overexpression causes a significant reduction of the adhesion ability [50]. Studies using dual-luciferase reporter assays showed that miR-143 directly binds to the 3′-UTR of GLI3. In addition, results from qRT-PCR and Western blot analysis demonstrated that the expression of miR-143 is negatively correlated to GLI3 and suppresses the migration of 5–8 F cells [51]. In the NPC samples, miR-375 expression is significantly reduced, while metadherin (MTDH) is significantly increased. NPC patients with high MTDH experience significantly lower survival, as well as higher distant relapse rates. Luciferase assays have also corroborated MTDH as a target of miR-375 [52]. miR-1 can induce NPC cell apoptosis by directly targeting the prothymosin alpha gene, in which siRNA and miR-1 accelerate the apoptotic process in cells treated with apoptosis inducers [53]. miR-124 is also commonly downregulated in NPC specimens and cell lines, and its expression is inversely correlated with clinical stages. Foxq1 is a novel direct target of miR-124, and its knockdown inhibits cell growth, migration, and invasion (Fig. 1) [54]. miR-138 is another downexpressed miRNA, and its ectopic expression suppresses NPC cell proliferation and colony formation, and also inhibits tumorigenesis in vivo (Fig. 1). Cyclin D1 is a direct target of miR-138, and its mRNA levels are inversely correlated with miR-138 expression [55]. miR-184, which is modulated by programmed cell death 4 (PDCD4), directly targets BCL2 and C-MYC and participates in PDCD4-mediated suppression of cell proliferation and survival in NPC [56].

### Onco-miRNAs are involved in the regulation of NPC

In tumorigenesis of NPC, some miRNAs called onco-miRNAs, function as oncogenes, for example, miR-18, miR-214, miR-155, and miR-141. miR-18, a member of the oncogenic miR-17-92 cluster, acts as an onco-miR in NPC development (Fig. 1). Indeed, miR-18a is upregulated in NPC samples and cell lines [57]. Clinical parameter studies showed that miR-18a levels are correlated with advanced stages of NPC, lymph node metastasis, EBV infection, and a higher death rate. miR-18a negatively regulates Dicer1 by binding to its 3′-UTR, resulting in global downregulation of miR-200 family and miR-143 expression. The EMT marker E-cadherin and oncogene K-Ras are aberrantly expressed after miR-18a transduction, and these alterations are directly induced by the downregulation of miR-200 family and miR-143 [58]. miR-18b directly suppresses the expression of connective tissue growth factor (CTGF) in NPC, and its downregulation is significantly associated with NPC progression and poor prognosis. In fresh clinical specimens, miR-18b was widely overexpressed and inversely correlated with CTGF expression [59]. miR-141 was also upregulated in the NPC specimens. Both c-Myc knockdown and the re-expression of the host defensive protein short palate, lung, and nasal epithelium clone 1 (SPLUNC1) can downregulate miR-141. The miR-141 inhibition, in turn, affects cell cycle, apoptosis, growth, migration and invasion. Studies using luciferase reporter assays have confirmed that bromodomain containing 3(BRD3), ubiquitin-associated protein 1 (UBAP1), and PTEN are the potential targets of miR-141 [60]. SPLUNC1 functions at the very early stage of NPC carcinogenesis. It regulates NPC cell proliferation, differentiation, and apoptosis through miR-141, which in turn regulates the expression of *PTEN* and *p27*. This signaling axis is negatively regulated by the EBV-coded gene *LMP*1 [61]. Although miR-155 is upregulated in the two EBV-negative NPC cell lines, Chinese nasopharyngeal carcinoma cell line 1 and nasopharyngeal carcinoma cell line from Taiwan (TW03), EBV-encoded LMP1, and LMP2A may further enhance miR-155 expression. Jumonji Domain 1A (JMJD1A) and BTB and CNC homology 1 (BACH1), putative targets of miR-155, can be downregulated by miR-155 mimics, while miR-155 inhibitor upregulates the expression of JMJD1A in the NPC cell lines. The downregulation of JMJD1A is significantly correlated with the N stage of the TNM classification, a lower 5-year survival rate, and a lower 5-year disease-free survival rate of NPC patients [62]. In addition, miR-155 expression is upregulated in EBV-positive NPC tissue samples and is correlated with plasma DNA copies of LMP1. The expression of miR-155 is also upregulated in the NPC cell lines when transfected with an LMP1-expressing plasmid. Upregulated miR-155 stimulates NPC cell proliferation, colony formation, cell migration, and invasion [63].

miR-214 is known to be overexpressed in NPC cell lines and tissues. miR-214 not only promotes NPC cell proliferation and invasion in vitro but also accelerates tumor formation and lung metastasis (Fig. 1). The expression of miR-214 is upregulated in NPC samples, especially in metastasis-prone NPC tumor tissues, while the lactotransferrin (LTF) expression level is negatively correlated with that of miR-214. These suggest that miR-214 targeting is partly responsible for the LTF downregulation in the NPC specimens [64]. The silencing of miR-214 by locked-nucleic-acid–anti-miR-214 in NPC cells results in the enhancement of apoptosis and suppression of in vitro cell proliferation and in vivo tumor growth in nude mice. Luciferase reporter assays have been performed to identify Bim as a direct target of miR-214 [65].

In addition to the above miRNAs, miR-421, miR-10b, miR-21, miR-30a, miR-93, miR-144, miR-146a, miR-149, miR-378, and miR-663 are also known to play oncogenic roles in NPC tumorigenesis (Fig. 1). Overexpression of miR-421 inhibits the forkhead box protein O4 (FOXO4) signaling pathway, and it downregulates p21, p27, Bim, and ligand (FASL) expression by directly targeting the 3′-UTR of FOXO4 [66]. miR-10b is highly expressed in EBV–LMP1-positive NPC cells, and its expression is downregulated by silencing LMP1 or Twist [67]. miR-10b mimics can promote the mobility and invasion of NPC cell lines, while miR-10b inhibitors impede this invasion. In addition, the expression of genes related to migration and invasion, such as *E-cadherin*, *vimentin*, and matrix metalloproteinase-9 *(MMP-9*), is different in CNE-2 cells transfected with miR-10b mimics or treated with miR-10b inhibitors. And miR-10b is found to upregulate *MMP-9*, while knockdown of miR-10b significantly downregulates *E-cadherin* expression [68]. miR-21 is an onco-miR, whose levels are elevated in the NPC tissues. Consequently, cell migration is notably inhibited by the downregulation of miR-21 in vitro, while miR-21 inhibitors can downregulate B cell CLL/lymphoma 2 (BCL2) expression. This suggests that BCL2 may be a target gene for the initiation and development of NPC [69]. The expression of miRNA-146a in human NPC was found to be elevated by EBV-associated antigen LMP1 probably through the activation of the miRNA-146a promoter [70]. A single-nucleotide polymorphism in miRNA-146a is associated with an increased risk of NPC [71]. miR-378 is commonly upregulated in both NPC tissues and NPC, although this is opposite to the reported results in plasma. Functional studies have shown that the upregulation of miR-378 dramatically promotes cell proliferation, colony formation, migration, and invasion in vitro, as well as tumor growth in vivo. Mechanistic investigations reveal that miR-378 overexpression can downregulate the expression of Transducer of ERBB2 (TOB2), a potential tumor suppressor, while miR-378 silencing can enhance the TOB2 expression [72]. miR-663 is found to be upregulated in NPC cells. Inhibition of miR-663 impairs the in vitro proliferation of NPC cells and the in vivo tumor growth of xenografts in nude mice. Mechanistically, miR-663 directly targets p21 (WAF1/CIP1) to promote cellular G1/S transition, since the inhibitory effects of miR-663 on the G1/S transition can be rescued by p21 (WAF1/CIP1) silencing [73].

### Metastatic miRNAs in NPC

To successfully metastasize, a tumor cell must accomplish a complex set of processes including invasion, survival, and arrest in the circulatory system, as well as colonization of foreign organs. Despite great advances in the knowledge of metastasis biology, the molecular mechanisms are not completely understood. Remarkably, a number of miRNAs exhibit a regulatory role in the metastatic program. miRNAs can promote or suppress various steps in the migration and metastasis of cancer cells [74], thereby affecting key steps such as EMT, migration, and angiogenesis (Fig. 1).

NPC exhibits invasive and metastatic features, and approximately 90 % of NPC patients show cervical lymph node metastasis at the time of initial diagnosis [75]. miR-93 directly targets transforming growth factor-β receptor II, thereby promoting NPC cell proliferation, invasion, and metastasis [76]. miR-378 dramatically promotes cell colony formation, migration, and invasion in vitro. miR-378 overexpression and silencing can downregulate or enhance the expression of the tumor suppressor TOB2, respectively [72]. miR-30a increases the capability of metastasis and invasion of the NPC tumor cells, both in vivo and in vitro, by targeting E-cadherin [77]. miR-155 stimulates NPC cell proliferation, colony formation, cell migration, and invasion by downregulating the expression of JMJD1A and BACH1 [63]. miR-144 is inversely correlated with the expression of tumor suppressor gene phosphatase and tensin homolog (PTEN) in the NPC specimens and cell lines; consequently, miR-144 suppresses the expression of PTEN to increase the expression of phosphorylated protein kinase B (pAkt) and cyclin D1, and promote G(1)-phase transition and then decreases the expression of E-cadherin to promote migration and invasion [78]. The expression of miR-149 is higher in NPC cell lines 5–8 F with high metastasis ability, and it promotes their mobility and invasion by downregulating the expression of E-cadherin [79].

Conversely, a few miRNAs serve as metastasis inhibitors; miR-200a inhibits the growth, migration, and invasion of C666-1 cells and conversely regulates EMT [39]. miR-9 is commonly downregulated in NPC [31]. Ectopic expression of miR-9 dramatically inhibits the proliferative, migratory, and invasive capacities of NPC cells in vitro and in vivo. The low plasma level of miR-9 is significantly correlated with increased lymphatic invasion and advanced TNM stage [32]. miR-9 also inhibits cell growth, migration, and invasion by regulating chemokine (C-X-C motif) receptor 4(CXCR4) expression [30]. miR-29c inhibits NPC cell migration and invasion in vitro and suppresses lung metastases in vivo by targeting TIAM1 [35].

### EBV-encoded miRNA in NPC

The herpes virus EBV is implicated in NPC and other human malignancies. EBV-encoded miRNAs are the first group of viral miRNAs to be identified. Although the close association of NPC with EBV infection has been known for more than four decades, the exact role of EBV in the pathogenesis of NPC malignancy remains unclear. Utilizing the deep sequencing technology to characterize the EBV miRNA transcriptome in clinical NPC tissues, more than 110,000 sequence reads and 44 Epstein–Barr virus encoded microRNA (EBV BART) were identified. The miRNA sequence analysis revealed that most of the highly abundant EBV miRNAs share their seed sequences (2–7 nt) with human miRNAs; it suggests that the seed sequence content may be an important factor underlying the differential accumulation of BART miRNAs [80]. NPC tumors are known to express a number of EBV-encoded proteins; they also express a large number of virus-encoded miRNAs, the most abundant of which are encoded from the BamHI-A region of the viral genome; they are thus called as BART miRNAs [81]. EBV-miR-BART1 is involved in regulating the metabolism-associated genes in NPC [82]. miR-BART17 is significantly more abundant in the plasma samples from NPC patients compared with those from non-NPC subjects [83]. miR-BART3 is abundantly expressed in NPC cells. The target site of miR-BART3 is located in the 3′-UTR of the determination of interleukin 4 commitment 1 (DICE1 ) tumor suppressor transcript, which is usually underexpressed in EBV-expressing NPC tissues [84]. The plasma levels of EBV-miR-BART7 are significantly higher in NPC patients compared with those from healthy individuals. The expression of EBV-miR-BART7 enhances the proliferation, migration, and invasion of NPC cells in vitro. Furthermore, NPC cells expressing EBV-miR-BART7 are more resistant to cisplatin [85]. miR-BART22 is highly expressed in the NPC tissues, and it may inhibit MAP3K5 expression, thereby reducing the phosphorylation of mitogen-activated protein kinase pathway downstream molecular and then inhibit NPC cell apoptosis and differentiation, and finally prevent the NPC cells from immune surveillance [86]. miR-BART9 is highly expressed in the NPC tissues and EBV-positive NPC cells but not in the normal tissues. Notably, the level of miR-BART9 expression was higher than that of miR-21 in eight of the nine NPC tissues examined.

### miRNAs as therapeutic targets or tools

Besides influencing practically all the biological processes including proliferation and apoptosis, miRNAs have great potential as therapeutic agents. Possibly, the most important advantage of miRNAs in comparison with conventional approaches that target single genes is their ability to target multiple molecules, frequently in the context of a network. This renders them extremely efficient in regulating distinct biological processes relevant to normal and malignant cell biology.

By miRNA plasmid constructed artificially, miRNA can effectively interfere with NPC cells by downregulating the expression of VEGF thus inhibiting the growth of tumor xenografts in vivo. Future application of miRNA in the gene therapy of NPC might be expected [87]. Forced expression of miR-125a-5p is known to enhance proliferation, migration, and invasion of HONE1 cells. NPC patients exhibit significantly higher expression level of miR-125a-5p than healthy controls. miR-125a-5p and curcumin are known to inhibit or upregulate the expression of tumor protein 53 (TP53), respectively. Taken together, these results indicate that curcumin exerts inhibitory effects on NPC by inhibiting the expression of miR-125a-5p and subsequently enhancing the expression of TP53 [88].

Low expression of miR-29c is positively correlated with therapeutic resistance in NPC patients; consequently, miR-29c substantially enhances the sensitivity of NPC cells to IR and cisplatin treatment by promoting apoptosis. It also represses the expression of anti-apoptotic factors, Mcl-1 and Bcl-2, in the NPC tissues and cell lines [36].

Gefitinib is found to inhibit the proliferation of two NPC cell lines in vitro and in vivo, wherein HK-1 cells are more sensitive to gefitinib than human nasopharyngeal carcinoma cell line 1 (HNE-1) cells. In addition, gefitinib treatment elevates the miR-125a-5p expression in the two cell lines and the serum of NPC tumor-bearing mice. This phenomenon is weak in the HNE-1 cells and strong in the HK-1 cells. Similarly, miR-125a-5p overexpression improves the anti-proliferative and pro-apoptotic effects of gefitinib on the NPC cells, while its downregulation abrogates these effects. MiR-125a-5p is also known to increase p53 protein expression in HNE-1 cells and decrease Her2 protein expression in HNE-1 and HK-1 cells [89].

Interestingly, miRNAs are known to influence cellular responses to specific therapy including radiotherapy. Radiosensitivity and radiation dose of X-ray can significantly affect the expression of miR-7 in the NPC cells, indicating that miR-7 plays an important role in the radioresistance of NPC cells. Thus, suppressed miR-7 expression may elevate the radiosensitivity of NPC cells [90]. miR-205, which is elevated in the radioresistant NPC cell line CNE-2R, is known to regulate the expression of PTEN, a tumor suppressor. Introducing miR-205 into CNE-2 cells suppresses PTEN protein expression following the activation of AKT; it increases foci formation and reduces post-irradiation apoptosis. On the other hand, knocking down miR-205 in CNE-2R cells compromises the inhibition of PTEN and increases cell apoptosis [91]. miRNA-324-3p contributes to the radioresistance of NPC by regulating the wingless-type MMTV integration site family, member 2B (WNT2B) signaling pathway. Thus, both miRNA-324-3p and WNT2B are potential biomarkers for NPC radioresistance [92].

### miRNAs as diagnostic tools

The need for clinical biomarkers for early diagnosis of NPC is extremely urgent; it should be considered that the survival and prognosis of NPC patients depends on the stages of the tumor at the time of detection. Some miRNAs that play an important role or highly express in NPC tumorigenesis may also serve as diagnostic or prognostic biomarkers of NPC. For example, most NPC patients with a poor outcome exhibit a high expression (>median) of miR-548q (70.6 %) and miR-483-5p (64.7 %) in their tissue samples, suggesting that miR-548q and miR-483-5p are potential biomarkers of NPC. Combining the receiver operating characteristic (ROC) analyses of these two miRNAs, an area under the ROC curve of 0.737 with 67.1 % sensitivity and 68.0 % specificity was obtained, which demonstrates the preliminary diagnostic value of plasma miRNAs [93]. The low level of plasma miR-9 is significantly correlated with increased lymphatic invasion and advanced TNM stage. Thus, plasma miR-9 could distinguish the locoregional from the metastatic NPC cases with high sensitivity and specificity. Furthermore, plasma levels of miR-9 are significantly elevated in post-treatment samples compared with pre-treatment samples. Thus, plasma miR-9 may serve as a useful biomarker to predict NPC metastasis and to monitor tumor dynamics [32]. NPC patients with low miR-483-5p and miR-103 expression have a better 5-year prognosis than those with a high expression; similarly, patients with a low concentration of miR-29a and let-7c have poorer prognosis [94]. Dicer and Drosha mRNA are significantly downregulated in NPC tissue specimens and cell lines when compared with controls, and the low expression of the Dicer and Drosha proteins is significantly correlated with shorter progression-free survival and overall survival (OS) of NPC patients [95].

Four serum miRNAs including miR-22, miR-572, miR-638, and miR-1234 were found to be differentially altered in NPC, and they were used to construct a miRNA signature. The combination of this miRNA signature and TNM stage was found to have a better prognostic value than the TNM stage or miRNA signature alone [96]. The miRNAs were found to be expressed differentially in the serum of NPC patients compared with the healthy controls. Based on these, a diagnosis equation with Ct difference method has been established to distinguish NPC cases from non-cancerous controls and validated for high sensitivity and specificity [97]. A signature of five miRNAs, each significantly associated with disease-free survival (DFS), was identified in the training set. A risk score from the signature and patients classified as high risk or low risk is calculated. Compared with the patients with low-risk scores, the patients with high risk scores in the training set have shorter DFS, distant metastasis-free survival, and OS [98]. Research from the Wang group indicated differential miRNA expression during tumor relapse, demonstrating the potential use of miRNAs in the classification of the repeated recurrence of NPC, beyond the histological approach [99].

## Conclusions

In summary, it is evident that miRNAs not only represent an additional level of complexity in the molecular portrait of NPC, contributing to tumor comprehension and subclassification, but also represent easily detectable biomarkers to predict prognosis and response to therapy. It is essential to validate their potential as biomarkers in different cohorts of samples, taking into account the evidence that profiling signatures are probably more statistically significant that single miRNAs in predicting outcomes. Moreover, even though the available, up-to-date data are almost exclusively from pre-clinical samples, miRNAs are promising therapeutic and diagnostic tools in NPC research.
